# GCSENet: A GCN, CNN and SENet ensemble model for microRNA-disease association prediction

**DOI:** 10.1371/journal.pcbi.1009048

**Published:** 2021-06-03

**Authors:** Zhong Li, Kaiyancheng Jiang, Shengwei Qin, Yijun Zhong, Arne Elofsson

**Affiliations:** 1 Department of Mathematical Sciences, School of Science, Zhejiang Sci-Tech University, Hangzhou, China; 2 Department of Biochemistry and Biophysics, Science for Life Laboratory, Stockholm University, Stockholm, Solna, Sweden; Icahn School of Medicine at Mount Sinai, UNITED STATES

## Abstract

Recently, an increasing number of studies have demonstrated that miRNAs are involved in human diseases, indicating that miRNAs might be a potential pathogenic factor for various diseases. Therefore, figuring out the relationship between miRNAs and diseases plays a critical role in not only the development of new drugs, but also the formulation of individualized diagnosis and treatment. As the prediction of miRNA-disease association via biological experiments is expensive and time-consuming, computational methods have a positive effect on revealing the association. In this study, a novel prediction model integrating GCN, CNN and Squeeze-and-Excitation Networks (GCSENet) was constructed for the identification of miRNA-disease association. The model first captured features by GCN based on a heterogeneous graph including diseases, genes and miRNAs. Then, considering the different effects of genes on each type of miRNA and disease, as well as the different effects of the miRNA-gene and disease-gene relationships on miRNA-disease association, a feature weight was set and a combination of miRNA-gene and disease-gene associations was added as feature input for the convolution operation in CNN. Furthermore, the squeeze and excitation blocks of SENet were applied to determine the importance of each feature channel and enhance useful features by means of the attention mechanism, thus achieving a satisfactory prediction of miRNA-disease association. The proposed method was compared against other state-of-the-art methods. It achieved an AUROC score of 95.02% and an AUPR score of 95.55% in a 10-fold cross-validation, which led to the finding that the proposed method is superior to these popular methods on most of the performance evaluation indexes.

## Author summary

Identifying miRNA-disease associations accelerates the understanding towards pathogenicity, which is beneficial for the development of treatment tools for diseases. Different from existing methods, our GCSENet captures the deep relationship between miRNA and disease through three heterogeneous graphs (disease, gene and miRNA) to promote an accurate prediction result. We performed the 10-fold cross validation to evaluate the performance of GCSENet, which can outperform many classic methods. Furthermore, we carried out case studies on four important diseases, which were used to evaluate the performance of our model regarding to the associations with experimental evidences in literature. The result shows that most predicted miRNAs (48 for lung neoplasms, 48 for heart failure, 48 for breast cancer and 50 for glioblastoma) in the top 50 predictions were confirmed in HMDD v3.0. As a result, it shows that GCSENet can make reliable predictions and guide experiments to uncover more miRNA-disease associations.

## Introduction

MicroRNAs (miRNAs) are a type of small non-coding RNAs (22~24 nucleotides in length), which function as an important regulator in human body [1]. MiRNAs have been proven to exert influence on such physiological processes as cell growth, cell differentiation, immune reaction by affecting gene expression after transcription. More and more studies have revealed that miRNA is closely related to human diseases, such as Parkinson’s disease [2] and cancer [3]. Therefore, the identification of association between miRNAs and diseases is of great significance for the study on disease pathogenesis and the development of drugs. However, it is costly and time-consuming to identify the associations between a pair of miRNA and disease/phenotype through biological experiment. Therefore, computational approaches have been adopted to predict these associations. The basic assumption is that the miRNAs associated with the same or similar diseases are more likely to be functionally related. The existing methods can be roughly divided into two categories: network based methods and machine learning based algorithms.

### Network based predictions

Jiang et al. [4] presented a miRNA-disease association (MDA) identification method through the hypergeometric distribution. However, it considered only the direct neighbors of each miRNA in the miRNA functional network and the number of overlapping genes while ignoring the functional connection between them. Liu et al. [5] introduced a computational model of random walk with restart for miRNA-disease association (RWRMDA) to identify new disease-related miRNAs, but it may be ineffective for new diseases without any known related miRNAs. By constructing a heterogeneous graph that integrated different types of heterogeneous biological datasets, Xuan et al. [6] proposed an algorithm of human disease-related miRNAs prediction (HDMP) to predict miRNA-disease associations based on the weighted *k* most similar neighbors. According to the miRNA-miRNA and disease-disease networks, Shi et al. [7] introduced a miRNA-disease association prediction method by constructing a gene layer between miRNA and disease, based on which the disease genes and miRNA target genes were taken as seeds for the calculation performed by random walk algorithm. Chen et al. [8] proposed another model called within and between score for miRNA-disease association prediction (WBSMDA) through integrating similarity for miRNAs and diseases, and combining within-score and between-score to obtain the final score for potential miRNA-disease association prediction. You et al. [9] proposed a novel path-based miRNA-disease association (PBMDA) prediction method to calculate the association score between miRNAs and diseases. Ji et al. [10] introduced a network embedding based heterogeneous information integration method by combining the known associations between protein, miRNA, lncRNA, disease and drug.

### Machine learning based predictions

Chen and Yan [11] proposed a regularized least squares method for the identification of miRNA-disease association. It applied a semi-supervised algorithm to identify the association between miRNAs and diseases. Xuan et al. [12] introduced two convolutional network based methods for predicting the candidate diseases. Based on the known miRNA-disease associations, Li et al. [13] proposed a matrix completion method for MDA (MCMDA). It applied the matrix completion algorithm to update the adjacency matrix of known miRNA-disease associations for the prediction of potential associations. Liang et al. [14] developed a novel method for identifying those disease-related candidate miRNAs based on adaptive multi-view multi-label learning. Peng et al. [15] adopted a regression model to obtain the feature input, before the transferral of it to convolutional neural network (CNN) for the final miRNA-disease association prediction result to be obtained by the supervised training.

Despite the effectiveness of above-mentioned methods in identifying MDA, there remain some challenges facing the improvement of prediction results. As for the network-based prediction, most of them are un-supervised ones solely based on networks without the involvement of labeled information. Recently, various relationships between miRNAs and diseases have been detected on the basis of biological experiment, which provides opportunities to predict the miRNA-disease association using the supervised model. Though some methods rely on convolutional neural networks to extract miRNAs and characterize diseases, they are incapable to capture the detailed structural information from their heterogeneous network, such as network topology and node neighborhood. Li et al. [16] introduced a method of nonlinear inductive matrix completion with graph convolutional network (GCN), but it is limited to extracting the feature from two heterogeneous networks of disease and miRNA, which means no consideration is given to the influence of gene network.

In this paper, a new prediction model integrating with GCN, CNN and SENet (GCSENet) was proposed to identify miRNA-disease association. Firstly, our consideration was given to the influence of gene network on the prediction of miRNA-disease association for the construction of a three-layer heterogeneous network containing disease, gene and miRNA. As this heterogeneous network possesses no regular spatial structure, GCN is applied to extract the features of disease-gene association and miRNA-gene association. Secondly, considering the influence of a gene on various diseases and miRNAs varies significantly when the disease-gene association and the miRNA-gene association are manifested, a feature weight for each disease-gene association and miRNA-gene association was set to reflect this difference. Thirdly, to distinguish the different importance of these two associations to determining the miRNA-disease association, a new feature component was constructed by combining the miRNA-gene association and the disease-gene association. Then, with regard to the characteristics of disease-gene association, miRNA-gene association and their combined association, the squeeze and excitation blocks of SENet were applied to determine the importance of each feature channel by means of the attention mechanism, and the re-calibration of the feature channel was realized through the weight matching. Finally, the fully connected layer and the softmax layer were set to make the final prediction of miRNA-disease association.

## Materials and methods

### Data

In this study, the interactions between miRNAs and diseases were captured using a heterogeneous network, which consists of a disease layer, a gene layer and a miRNA layer. It also involves data with the experimentally validated miRNA-disease associations, disease-gene associations, miRNA-gene associations, disease semantic similarity, miRNA functional similarity, disease network, gene network and miRNA network.

On the basis of miRNA-disease prediction tasks, our model was also verified on the phenotype of human disease and miRNA prediction. It contains the phenotype network, gene network and miRNA network. The gene network and miRNA network in the miRNA-phenotype task are the same as those used for the miRNA-disease prediction. The association data involves miRNA-phenotype associations, phenotype-gene associations and miRNA-gene associations. In order to construct the phenotype network, it entails the calculation of phenotype similarity as well.

### Human miRNA-disease associations, disease-gene associations and miRNA-gene associations

The known human miRNA-disease associations were obtained from the experimentally verified miRNA-disease database HMDD v3.0 [17], including 1206 miRNAs, 893 diseases and 32281 experimentally supported miRNA–disease association entries. The association of miRNA-disease is indicated in the form of adjacency matrix *Y*, where *Y*(*i*,*j*) = 1 denotes a miRNA *m*_*i*_ is associated with a disease *d*_*j*_, *Y*(*i*,*j*) = 0 means the association between them is either unknown or unobserved.

The disease-gene associations were captured from DisGeNET v7.0 database [18]. It integrates disease-gene associations from expert curated repositories. Only the manually confirmed disease-gene associations were used in our experiment. The link to the data is http://www.disgenet.org/.

As a comprehensive archive, miRWalk2.0 [19] provides the largest available collection of both predicted and experimentally verified miRNA-gene associations. The miRNA-gene associations were identified by inputting the target miRNA to obtain the associated gene from this database.

### Disease semantic similarity

The Mesh database (http://www.ncbi.nlm.nih.gov/) is available for conducting analysis of the relationship between different diseases. It divides all of the diseases into different categories. Our hierarchical directed acyclic graph *DAG*(*T*(*D*), *E*(*D*)) was constructed directly on the basis of Mesh. Herein, *T*(*D*) denotes the disease node set, including disease *D* and its ancestor nodes, *E*(*D*) indicates the directly connected set of all father nodes and child nodes in this set, representing the relationship between different diseases, then the semantic contribution of a disease *d* to the disease *D* is denoted by *D*_*d*_(*d*)

{Dd(d)=1ifd=DDd(d)=max{Δ⋅Dd(d˙)|d˙∈childrenofd}ifd≠D (1)

$$DV(D)=\sum_{d\in T(D)}D_{d}(d)$$ (2)

where Δ is the semantic contribution decay factor, which shows that as the distance between disease *D* and its ancestor diseases increase, their contribution to the semantic value of disease *D* diminishes on a continued basis. Accordingly, the contribution to the semantic value of disease *D* itself is defined as 1 when disease *D* is located in the 1-th layer. The contribution of its ancestor disease is supposed to be multiplied by the semantic contribution decay factor. Usually, Δ value is set as 0.5 [8,20]. *DV*(*D*) represents the contribution value as obtained by disease *D*. Based on this assumption, the semantic similarity between two diseases *d*(*i*) and *d*(*j*) can be calculated as

$$S(d(i),d(j))=\frac{\sum_{t\in T(d(i))\cap T(d(j))}\left(D_{d(i)}(t)+D_{d(j)}(t)\right)}{DV(d(i))+DV(d(j))}$$ (3)

### MiRNA functional similarity

MiRNA functional similarity was obtained by the associated diseases. Firstly, the maximum similarity S(*d*_*t*_, *D*_*T*_) between one disease *d*_*t*_ and one group of diseases *D*_*T*_ was calculated by

$$S(d_{t},D_{T})=\underset{1\leq i\leq k}{\max}(S(d_{t},d_{t_{i}}))$$ (4)

where S(*d*_*t*_, *D*_*T*_) is the similarity between disease *d*_*t*_ and the disease set $D_{T}=\{d_{t_{1}},d_{t_{2}},\ldots ,d_{t_{k}}\}$, $d_{t_{i}}$ is one of the diseases in *D*_*T*_, *k* is the number of diseases.

For the similarity of two miRNAs, consideration was given to the impact of each disease in its corresponding disease set, which is expressed as

$$MISIM(m_{1},m_{2})=\frac{\sum_{1\leq i\leq m}s(d_{t_{i}},D_{T_{2}})+\sum_{1\leq j\leq n}s(d_{t_{j}},D_{T_{1}})}{m+n}$$ (5)

where $D_{T_{1}}$ is the disease set associated with *m*_1_, $D_{T_{2}}$ is the disease set associated with *m*_2_, *m* is the number of diseases in $D_{T_{1}}$, *n* is the number of diseases in $D_{T_{2}}$.

### Disease network, gene network and miRNA network

The disease similarity network was obtained from You et al. [9]. The diseases having no associated genes in the gene network were removed. The interactions of genes in STRING [21] were manually extracted from the literature by the expert biologists responsible for reading, interpreting and analyzing the published data. The gene network was downloaded from STRING database (https://string-db.org/cgi/download). To construct the miRNA network, a miRNA similarity matrix generated by the miRNA similarity (MISIM) database (http://www.cuilab.cn/files/images/cuilab/misim.zip) was applied.

### Phenotype network

The phenotype network was constructed using the traditional Resnik method [22] through Human Phenotype Ontology (HPO) [23], which was also used by Masino et al. [24]. The specific information can be obtained from https://hpo.jax.org.

### Human miRNA-phenotype associations, phenotype-gene associations and miRNA-gene associations

The data on miRNA-phenotype was obtained from miRWalk2.0 database [19]. It is based on the published and validated experimental data (http://zmf.umm.uni-heidelberg.de/apps/zmf/mirwalk2/index.html). Phenotype-gene associations were obtained from HPO database (https://hpo.jax.org/app/download/annotation). MiRNA-gene associations were the same as what were used in the miRNA-disease association task.

### Phenotype similarity

The HPO also provides a standardized vocabulary of phenotypic abnormalities (phenotypes) encountered in human disease. Let *p*_1_ and *p*_2_ be two phenotypes, and *S* be the set of all common ancestors of *p*_1_ and *p*_2_. *p*_*m*_ indicates the phenotype that has a minimum of gene annotations in *S*. The similarity between *p*_1_ and *p*_2_ can be defined as

$$sim(p_{1},p_{2})=-log\frac{N_{P_{m}}}{N}$$ (6)

where $N_{P_{m}}$ represents the number of gene annotations of *p*_*m*_, including the gene annotations of its descendants, and *N* refers to the total number of genes involved in HPO. Only the phenotype terms that have a minimum of one gene involved in the gene network were selected. The human phenotype ontology and annotation data can be obtained from https://hpo.jax.org/app/.

**Fig 1:**
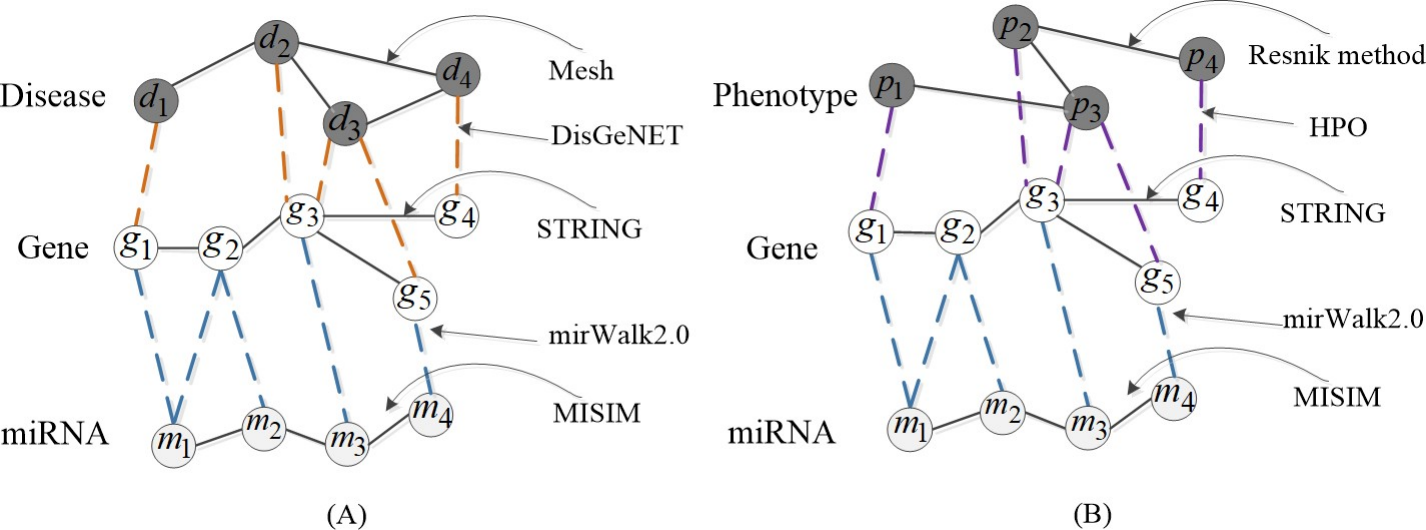
(A) shows the composition of the three-layer heterogeneous network (disease-gene-miRNA network), where yellow lines represent disease-gene connections, blue lines mean miRNA-gene connections. (B) shows the phenotype-gene-miRNA network, where purple lines represent phenotype-gene connections and blue lines mean miRNA-gene connections.

The disease semantic similarity network, gene network, miRNA functional similarity network, experimentally valid miRNA-disease, disease-gene, and miRNA-gene interactions were combined to obtain the whole disease-gene-miRNA heterogeneous network as illustrated in Fig 1A. Similarly, the phenotype-gene-miRNA network is shown in Fig 1B.

The association types include disease-gene associations from DisGeNET v7.0, phenotype-gene associations from HPO, miRNA-gene associations from miRWalk2.0, gene-gene associations from STRING, and the number of associations are 10283, 29511, 5114, 19237, respectively.

## GCSENet prediction model

**Fig 2:**
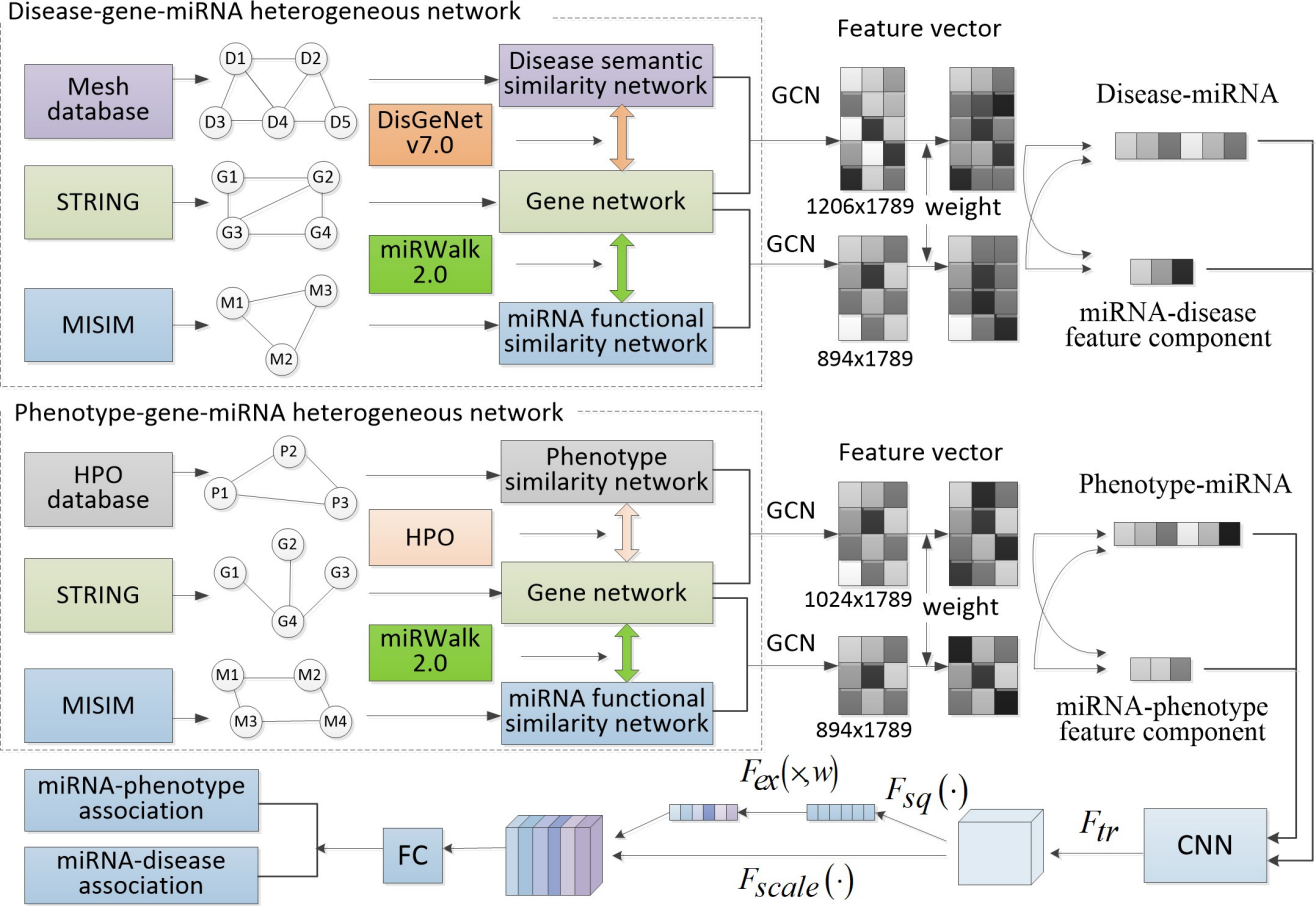
The framework of GCSENet.

Our miRNA-disease association prediction model is comprised of three parts as shown in Fig 2. The first one is the extraction of interaction feature based on GCN from the disease-gene-miRNA three-layer structure. The second one sets weight for the features and adds new feature components to refine the features. The third one applies CNN combined with SENet to make the prediction of miRNA-disease associations.

### Feature generation

Based on the three-layer heterogeneous network (disease, gene and miRNA), the graph feature of disease-gene and miRNA-gene networks was extracted respectively through GCN [25,26] to obtain the disease-gene feature components and miRNA-gene feature components, before the use of them for generating the disease and miRNA feature vector.

Herein, *G* = (*υ*, *e*) was used to represent the structure of a graph, where *υ* represents the set of nodes and *e* indicates the set of connected edges. The adjacency matrix is denoted as *A*, and the feature information of the nodes is indicated by $x_{i}\in {\mathbb{R}}^{m_{i}}$, *i* ∈ *υ*, where the value of *m*_*i*_ is different depending on the exact type of nodes *i* (disease, gene or miRNA). Thus, the embedding processing was adopted to make the features equivalent to *z*_*i*_ ∈ ℝ^*c*^, *c* ≪ *m*_*i*_, with a unified dimension.

In our disease-gene and miRNA-gene heterogeneous network, GCN aggregates and transforms the feature information from the node feature, node neighborhood and network topology. The architecture of signal transmission is shown as follows

$$h_{i,k}=\sum_{l}\sum_{j\in N_{i}^{l}}c_{i,j}W_{l}^{k}z_{j,k}+W_{t_{i},s}^{k}z_{i,k}$$ (7)

$$z_{i,k+1}=\phi (h_{i,k})$$ (8)

where $z_{i,k}\in {\mathbb{R}}^{c_{k}}$ is the embedding feature of the node *i* (disease, gene and miRNA) in the *k*-th graph convolutional layer, *c*_*k*_ is the element dimension of the hidden layer. *h*_*i*,*k*_ represents the feature vector output through the *k*-th hidden layer. $W_{t_{i},s}^{k}$ is the weight parameter of the node to itself, *t*_*i*_ represents the type of node. $N_{i}^{l}$ is the neighborhood of node *i*, *l* is the type of connection (gene-gene, disease-disease, miRNA-miRNA, disease-gene, miRNA-gene). $W_{l}^{k}$ is a weight parameter of type *l*, such as $W_{dd}^{k}$, $W_{gd}^{k}$, $W_{gg}^{k}$, $W_{dg}^{k}$, $W_{mg}^{k}$, $W_{mm}^{k}$. *c*_*i*,*j*_ is the normalization parameter [26], *ϕ* is the non-linear activation function, here refers to linear unit ReLu [27].

Above is the calculation formula for a node. It is worth noting that the aforementioned aggregation and transformation formulas are related to the neighbors of a certain node, which means the computational graph architecture can show the difference for nodes depending on the structure of local neighborhood. However, the parameters $W_{l}^{k}$ and $W_{t_{i},s}^{k}$ are related solely to the connection type, rather than the node neighborhoods, which means these parameters can be shared throughout the graph.

Finally, $z_{i,N^{+}}$ was used to represent the last embedding feature of the *i-*th node, where *N*^+^ denotes the number of layers involved in graph convolution.

After the embedding feature was obtained by GCN, the relationship between disease-gene pairs and miRNA-gene pairs was established. The association between disease *d*_*i*_ and gene *g*_*j*_ as well as the association between miRNA *m*_*k*_ and gene *g*_*j*_ can be expressed as

$$p(d_{i},g_{j})=\sigma (z_{d_{i}}^{T}W_{dg}z_{g_{j}})$$ (9)

$$p(m_{k},g_{j})=\sigma (z_{m_{k}}^{T}W_{mg}z_{g_{j}})$$ (10)

where $z_{d_{i}}^{T}\in {\mathbb{R}}^{c}$ is the embedding feature of the disease node *d*_*i*_ obtained by learning, $z_{g_{j}}\in {\mathbb{R}}^{c}$ is the embedding feature of the gene node *g*_*j*_. *W*_*dg*_ ∈ ℝ^*c*×*c*^ is a trainable parameter matrix representing the modeling of the interaction between diseases and genes. *W*_*mg*_ ∈ ℝ^*c*×*c*^ is a trainable parameter matrix representing the modeling of the interaction between miRNAs and genes. σ is the sigmoid activation function which controls the association in the range of (0,1).

### Feature processing

**Fig 3:**
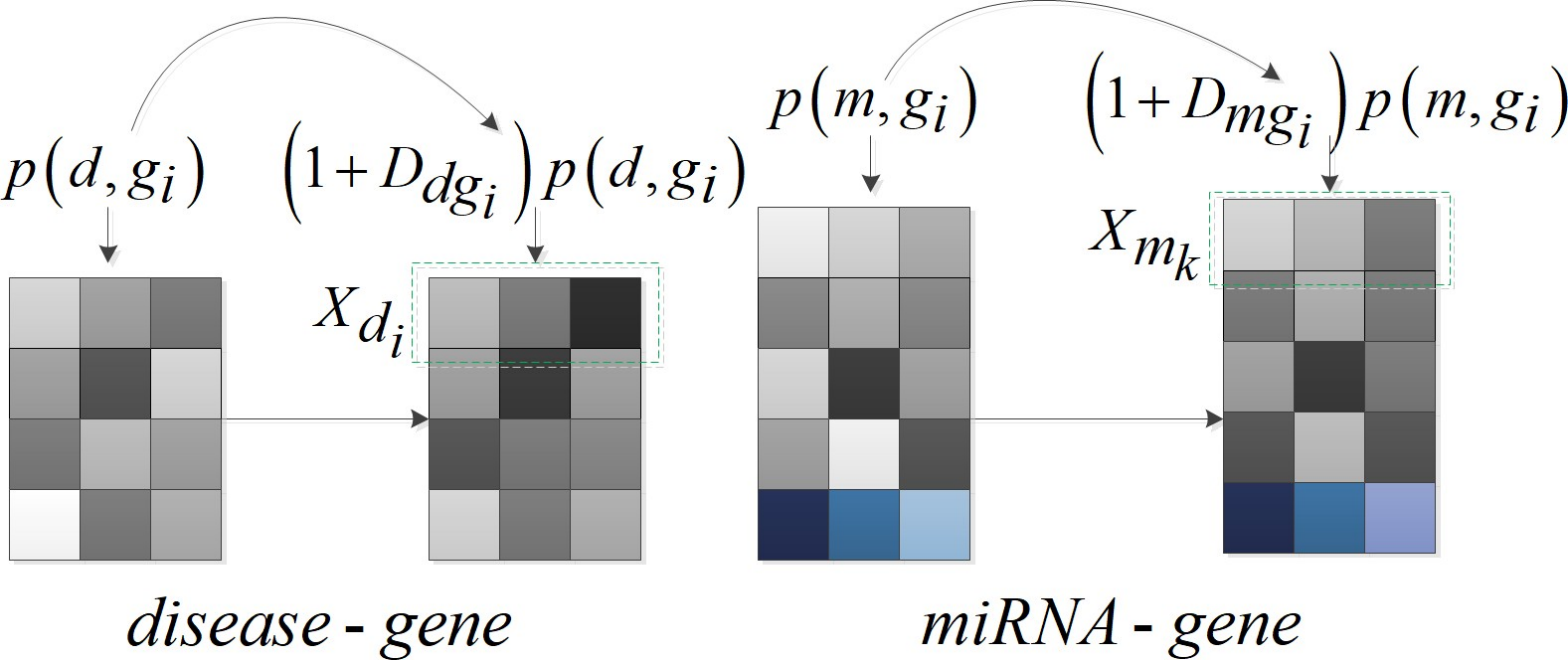
Weighted feature processing for disease-gene and miRNA-gene.

Since the number of diseases and miRNAs connected to the genes leads to different impacts on the disease-gene and miRNA-gene associations, the weighting operation was further conducted to treat the relationship between disease-genes and miRNA-genes in (9) and (10), which is illustrated in Fig 3. It was assumed that a gene connected to only one disease or linked to many diseases represents different cases, and the contribution from a gene to one disease or multiple diseases is different [9]. Here, a weighting method was applied for the feature vectors of miRNA-gene and disease-gene associations, namely, we introduced the parameter *D* which is set as the reciprocal of the number of diseases or miRNAs linked to a gene to reflect the different contribution. The following formula was applied to update the feature between disease and gene $X_{d_{i}}$ as well as the feature between miRNA and gene $X_{m_{k}}$

$$X_{d_{i}}=[(1+D_{dg_{1}})p(d_{i},g_{1}),\ldots ,(1+D_{dg_{n}})p(d_{i},g_{n})]$$ (11)

$$X_{m_{k}}=[(1+D_{mg_{1}})p(m_{k},g_{1}),\ldots ,(1+D_{mg_{n}})p(m_{k},g_{n})]$$ (12)

where $D_{dg_{i}}=\{\begin{array}{c} 0,deg(dg_{i})=0\\ \frac{1}{deg(dg_{i})},deg(dg_{i})\neq 0 \end{array}$, *deg*(*dg*_*i*_) denotes the number of diseases connected to gene *g*_*i*_, $D_{mg_{i}}=\{\begin{array}{c} 0,deg(mg_{i})=0\\ \frac{1}{deg(mg_{i})},deg(mg_{i})\neq 0 \end{array}$, *deg*(*mg*_*i*_) is the number of miRNAs connected to gene *g*_*i*_.

**Fig 4:**
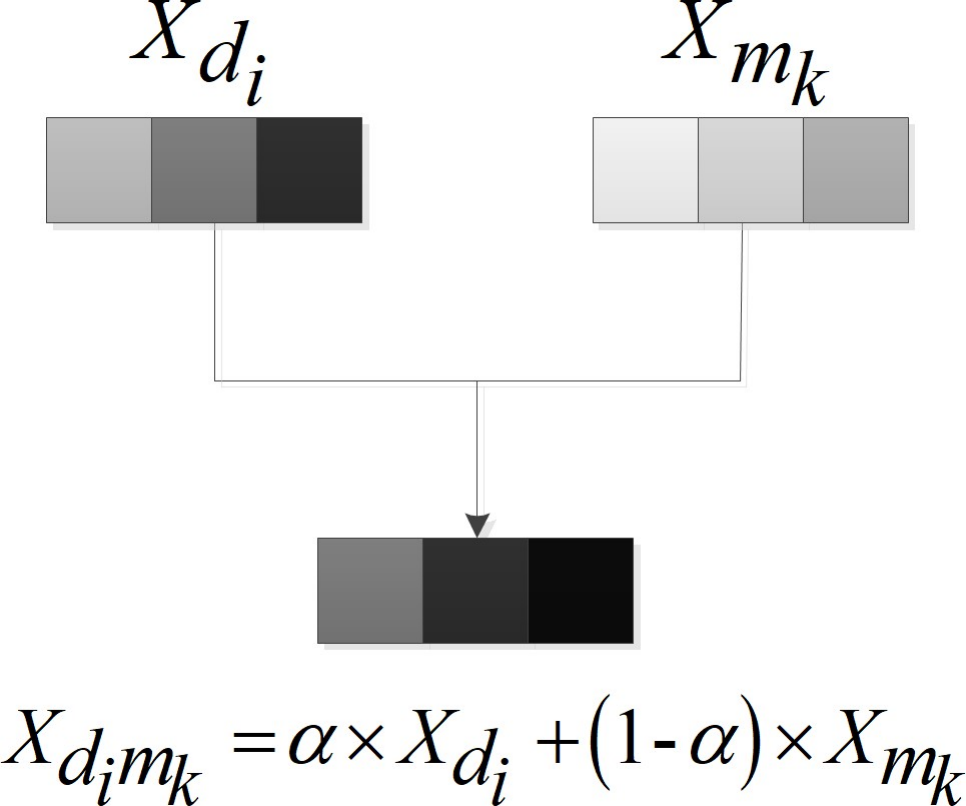
New feature component addition.

Besides, it was considered that the disease-gene and the miRNA-gene relationships play a different role as well in determining whether miRNA-disease association is existent. Therefore, a new feature component $X_{d_{i}m_{k}}=\alpha \times X_{d_{i}}+(1-\alpha )\times X_{m_{k}},\alpha \in (0,1)$, α ∈ (0,1), was introduced to reflect this difference, as shown in Fig 4. Finally, $\left[X_{d_{i}},X_{m_{k}},X_{d_{i}m_{k}}\right]$, *i* ∈ *υ*(*d*), *k* ∈ *υ*(*m*) was formed as the complete feature input of the neural network.

### Neural network framework

Our neural network framework includes GCN, CNN and SENet. Firstly, the features of three heterogeneous networks of miRNA, gene and disease were extracted through GCN. After the normalization of the above-mentioned complete features, these features were inputted to the convolution layer in CNN, and the feature channels were assigned the weight through SENet. Then, the pooling layer was used to reduce the dimensionality of features. Finally, the fully connected layer and the softmax layer were set to make the final prediction.

The final form of graph convolutional neural network is

$$H^{\left(l+1\right)}=\sigma ({\tilde{D}}^{-\frac{1}{2}}\tilde{A}{\tilde{D}}^{-\frac{1}{2}}H^{\left(l\right)}W^{\left(l\right)})$$ (13)

where *H*^(*l*)^ ∈ ℝ^*N*×*D*^ is the input of *l*-th GCN network, the initial input is *H*^(0)^ = *X*, *N* is the number of nodes in the graph, each node is represented by a *D*-dimensional feature vector. $\tilde{A}$ = A + *I*_*N*_ is the adjacency matrix with added self-connections, $\tilde{D}$is the degree matrix ${\tilde{D}}_{ii}=\sum_{j}{\tilde{A}}_{ij}$. *W*^(*l*)^ ∈ ℝ^*D*×*D*^ is the parameter matrix that needs training, σ is the activation function.

Then, the CNN combined with SENet block [28] was chosen to process the feature information. The SENet network is equivalent to an embedding structure used to weight features on the channel level. It consists of two parts, one of which is Squeeze. Through this operation, the information related to the feature space was averaged to a value for obtaining the global feature on the channel level

$$z_{c}=F_{sq}(u_{c}=\frac{1}{W\times H}\sum_{i=1}^{W}\sum_{j=1}^{H}u_{c}(i,j))$$ (14)

The second part is Excitation, which is realized by two fully connected layers. The first full connection layer compresses *C* channels into *C*/*r* channels to reduce the amount of calculation, and the second one recovers *C* channels, where *r* is the compression ratio. In this operation, the model learns the correlation between channels before assigning the learned channel correlation coefficient to each channel. This mechanism is effective in making the model pay more attention to the channel features with essential information, thus suppressing those insignificant channel features

$$s=F_{ex}(z,W)=\sigma (g(z,w))=\sigma (W_{2}\delta (W_{1}z))$$ (15)

Finally, the maximum pooling layer was applied to downsample the intermediate features. The result obtained after pooling was inputted to our last two layers (the fully connected layer and the softmax layer) for the final classification.

We have verified the effects of GCN module, feature weights, new feature component and SENet module, and found each component is beneficial to our prediction model, which are described in the following section of comparison with different GCSENet components.

### Experiment settings

In our GCSENet model, a three-layer network in GCN was adopted for the framework setting. For the miRNA-disease prediction, the compression factor *r* of SENet is 4, the dropout percentage equals 0.5. The L_2_ regularization parameter is 0.002. α is set as 0.57. With 64 samples included in every batch, 150 epochs were run in the miRNA-disease prediction task. For the miRNA-phenotype prediction, α is set as 0.5, the batch size is 128 and the number of epochs is 20. For both of the prediction tasks, the Adam optimization algorithm with an initial learning rate 1*e* − 2 was used to optimize the cross-entropy loss. The initial parameter is set according to the references Peng et al. [15] and Hu et al. [28]. All parameters are obtained by the grid search method.

We evaluate the performance of GCSENet and other five methods (i.e., WBSMDA [8], PBMDA [9], MDACNN [15], SAEMDA [10], NIMCGCN [16]) on the task of predicting miRNA-disease associations. As for training, the data is obtained from five datasets (Mesh, DisGeNet, STRING, miRWalk2.0 and MISIM). We train our model and other methods on these datasets for the same setting. As for testing, the data is chosen from benchmark2019 dataset [29] as the independent test set to make a relatively fair comparison.

Due to the absence of negative samples, we randomly generate a negative set with the same size as the positive set. For a particular disease, the positive sample is set as the relationship of miRNA-disease recorded in benchmark2019 dataset, and the connection of miRNA-disease not in benchmark2019 dataset is set as the negative sample. Note that some of the negative associations may actually be positive but unrecorded in the database. For the prediction of miRNA-phenotype association, the positive set was chosen from miRWalk2.0 [19], and the negative samples were generated in the similar way. Besides, we set a different ratio of negative and positive samples to verify the robustness of our method.

We use the 10-fold cross validation (CV) to validate our framework. The setting follows some principles: firstly, homologous genes would need to be placed into a single fold, so that they are not mixed between folds. Secondly, all relationships with particular disease should be put into separate folds. Thirdly, potentially diseases involved with similar relationships would also need to be stratified into training and testing.

### Performance evaluation

The area under the receiver operating characteristic curve (AUROC) and the area under precision-recall curve (AUPR) were taken as the main evaluation metrics [30]. The prediction results were divided into four categories [31], including true positive (TP), false negative (FN), false positive (FP), and true negative (TN). TP refers to a positive group samples that are correctly predicted, FN indicates a positive group that is incorrectly predicted to be negative, FP denotes a negative group that is incorrectly predicted to be positive and TN refers to a correctly predicted negative group. Based on these indexes, the following evaluation criteria were applied to project the performance of our model for the comparison against other methods. Precision is defined as the proportion of the correct number of positive samples in the total number of samples determined by the classifier as positive. Recall is referred to as the proportion of the correct number of positive samples in the actual number of positive samples. F1-score is the harmonic mean of the precision and recall.


$$\text{precision=}\frac{\text{TP}}{\text{TP+FP}}$$ (16)

$$\text{recall=}\frac{\text{TP}}{\text{TP+FN}}$$ (17)

$$\text{F}1-\text{score}=2\cdot \frac{\text{precision}\cdot \text{recall}}{\text{precision}+\text{recall}}$$ (18)

### Experimental results and discussion

To demonstrate the effectiveness of each part in our GCSENet framework, the GCSENet model was compared with four different versions, each of which had a different setting in feature acquisition, feature weighting, feature component adding and SENet processing. Then, the prediction results were compared between our model and other state-of-the-art methods. Finally, GCSENet was applied to the prediction of four specific diseases for validating our model.

### Comparison with different GCSENet components

The prediction result was first compared between the complete GCSENet model and the model without the feature extraction by GCN (extracted by the regression model), the model without weighting the feature, the model without adding the feature component and the model without using SENet, respectively. The experimental results are presented in Table 1.

**Table 1:** Prediction performance comparison with different GCSENet components.

Different GCSENet components	AUROC	AUPR	Precision	Recall	F1-score
Complete GCSENet	0.9502	0.9555	0.8795	0.8494	0.8642
Without GCN (regression model)	0.8886	0.8889	0.8199	0.7925	0.8059
Without weighting	0.8878	0.9050	0.8299	0.7270	0.7751
Without Feature component	0.8508	0.8419	0.8156	0.7883	0.8017
Without SENet	0.9308	0.9162	0.8536	0.8407	0.8471

**Fig 5:**
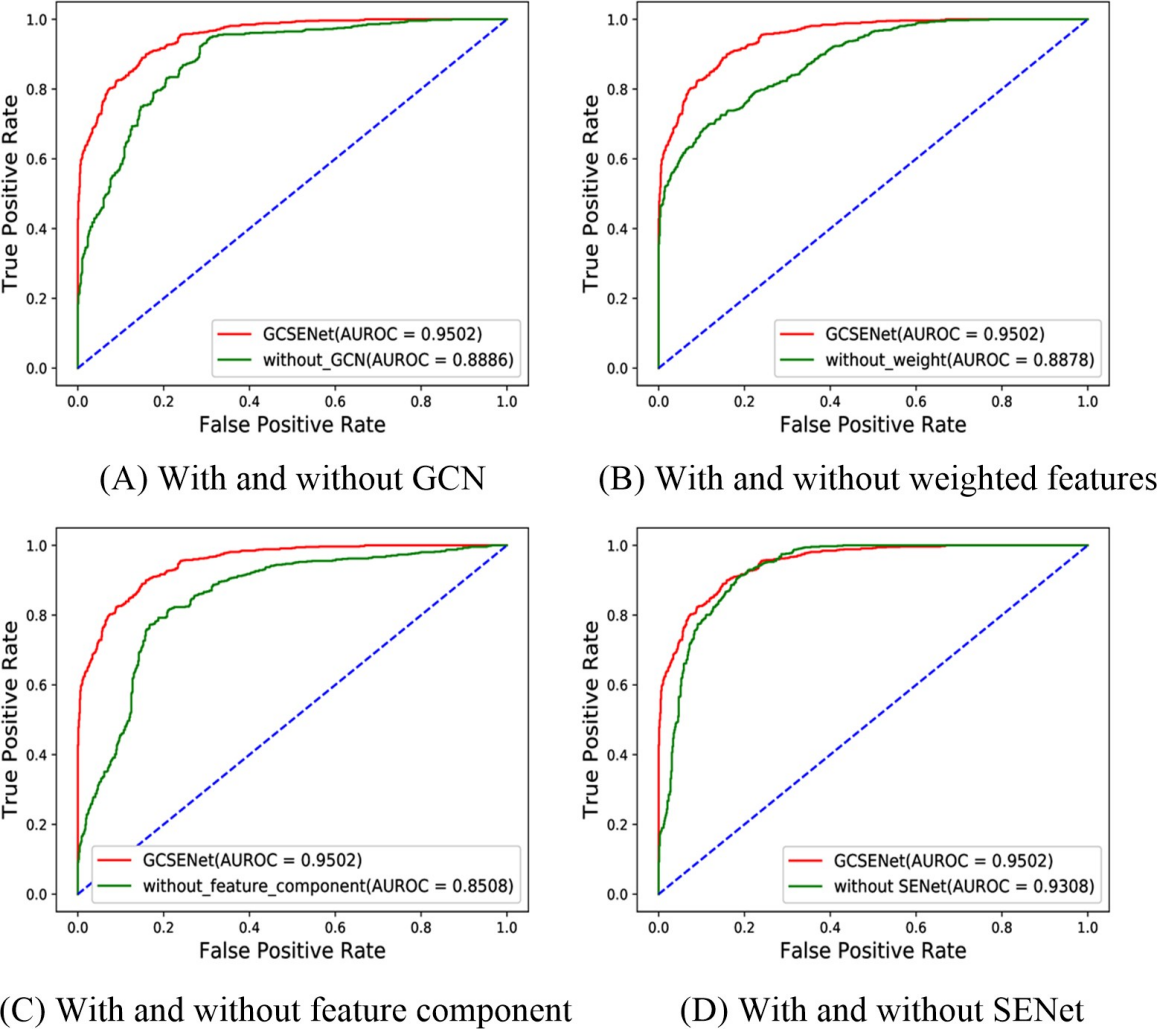
Comparison of ROC curves with different GCSENet components.

To reflect the advantage created by using GCN to extract structural features of heterogeneous networks, the features without using GCN and extracted by the regression model [15] were taken as a comparison for experiments (Fig 5A). It was found out that the AUROC by the regression feature reached 0.8886 while our result by the feature using GCN can realize 0.9502. For other evaluation indexes shown in Table 1, the results by the features extraction using GCN were higher than those by the regression model. Especially on AUPR and Precision, the increase exceeded about 7% and 6%, respectively, indicating that GCN can produce a better feature extraction effect for irregular network structures (disease, gene and miRNA).

Since the relationship in disease-gene tends to be affected by the number of diseases linked to each gene (similarly, the relationship in miRNA-gene is affected by the number of miRNAs linked to each gene), the weight was assigned to the relationship between disease-gene and miRNA-gene depending on the number of diseases and miRNAs associated with the gene. A comparison was performed between the original disease-gene and miRNA-gene features and the features by assigning these weights. The ROC curve is shown in Fig 5B and the results of main evaluation indexes are indicated in Table 1. It was found out that there was a close 7% improvement of AUROC, a 12% improvement of Recall and 9% increase of F1-score by the weighted features, respectively, suggesting that as the contribution of gene to related diseases and miRNAs varies, the use of weighting coefficient based on the number of miRNAs and diseases connected to genes is effective in reflecting the level of diversity.

Though the feature of miRNA-disease associations involves two parts (miRNA-gene and disease-gene), they play different roles in determining miRNA-disease association. Therefore, a new feature component was introduced for the prediction, namely, an association combination of miRNA-gene and disease-gene. Fig 5C shows the ROC comparison result. From Table 1, it can be seen that the result without using new feature component can reach merely 0.8508 on AUROC, which is 10% lower than if our method was applied. For the AUPR index, it was also 11% lower compared to our method. These results demonstrate that the new feature component, which is applied to integrate the miRNA-gene and disease-gene association proportions, fits well on determining the relationship between disease and miRNA.

Due to the inspiration derived from the attention mechanism [32], SENet was used to refine the feature on channels for enhancing the effectiveness of features in the spatial dimension. In the training process, it can increase the proportion of important feature channels, while weakening the relatively insignificant channels. Therefore, the features inputted by our model are more representative and effective in improving the training outcome in the neural network. It was found out that there was a roughly 2% improvement of the AUROC compared in Fig 5D. Moreover, there was an increase of major evaluation indexes, as shown in Table 1. Through the feature recalibration performed by SENet, it allowed the model to produce a better screening effect on the features, as a result of which an excellent prediction performance can be made achievable.

### Performance comparison with different methods

**Fig 6:**
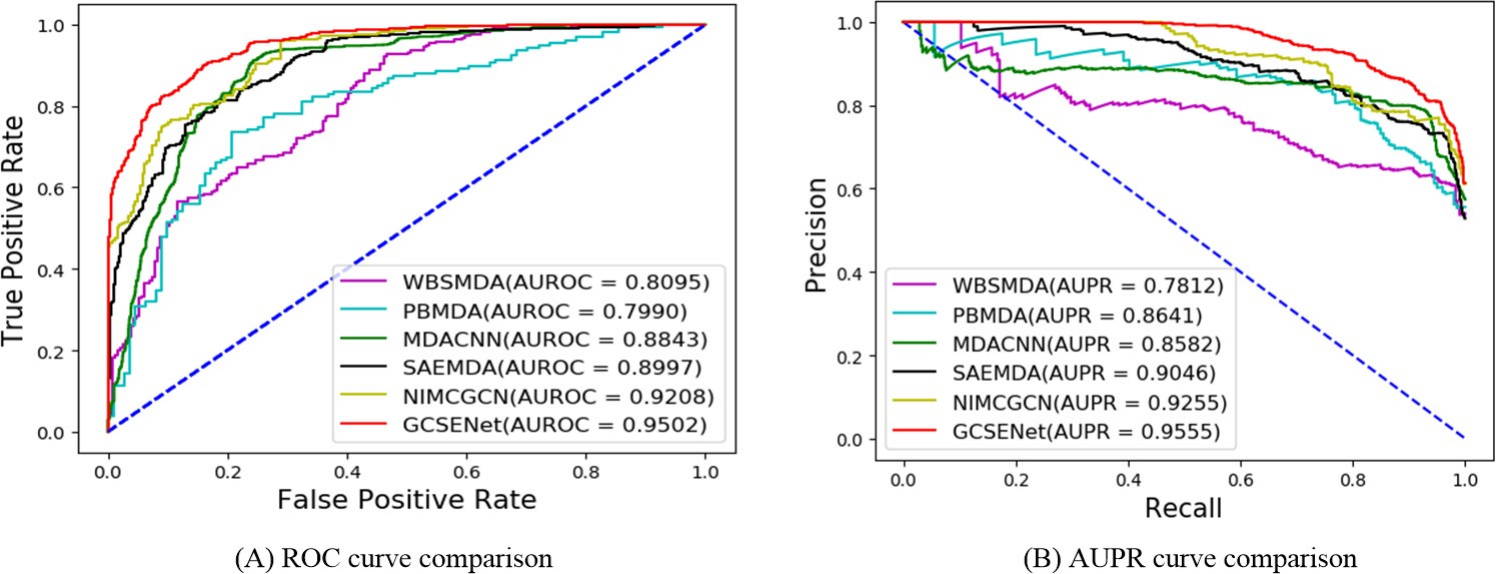
Comparison of different ROC and AUPR curves in miRNA-disease association prediction.

Additionally, the performance of our GCSENet model was compared against that of other popular methods including WBSMDA [8], PBMDA [9], MDACNN [15], SAEMDA [10] and NIMCGCN [16] for the prediction of miRNA-disease associations. The results are shown in Table 2, which reveals that GCSENet achieved the most excellent performance among all methods in terms of AUROC, AUPR, Precision, Recall and F1-Score, respectively. For example, the average AUROC achieved by GCSENet across the 10-fold cross validation was 0.9502, which is evidently higher compared to other five methods (average AUROC values of WBSMDA, PBMDA, MDACNN, SAEMDA, NIMCGCN are 0.8095, 0.7990, 0.8843, 0.8997 and 0.9208, respectively). Fig 6A shows the comparison of different ROC curves. As for the AUPR score, our GCSENet model reached 0.9555, which is about 0.03 higher than the second best NIMCGCN method. Fig 6B shows the comparison of different AUPR curves. For the Precision and Recall scores, our method showed an increase of 5% and 4% compared to MDACNN, and had an increase of 6% and 2% compared to NIMCGCN. For the F1-score, GCSENet also achieved the highest goal of 0.8642 among these methods. In summary, our method achieved an improvement to the indicators of miRNA-disease prediction tasks compared to these latest methods.

**Table 2:** Performance comparison with typical methods for miRNA-disease association prediction.

methods	AUROC	AUPR	Precision	Recall	F1-score
WBSMDA	0.8095	0.7882	0.6667	0.7671	0.7134
PBMDA	0.7990	0.8361	0.7778	0.7925	0.7850
MDACNN	0.8843	0.8823	0.8298	0.8060	0.8177
SAEMDA	0.8997	0.9046	0.8500	0.7577	0.8012
NIMCGCN	0.9208	0.9287	0.8112	0.8256	0.8183
GCSENet	0.9502	0.9555	0.8795	0.8494	0.8642

**Fig 7:**
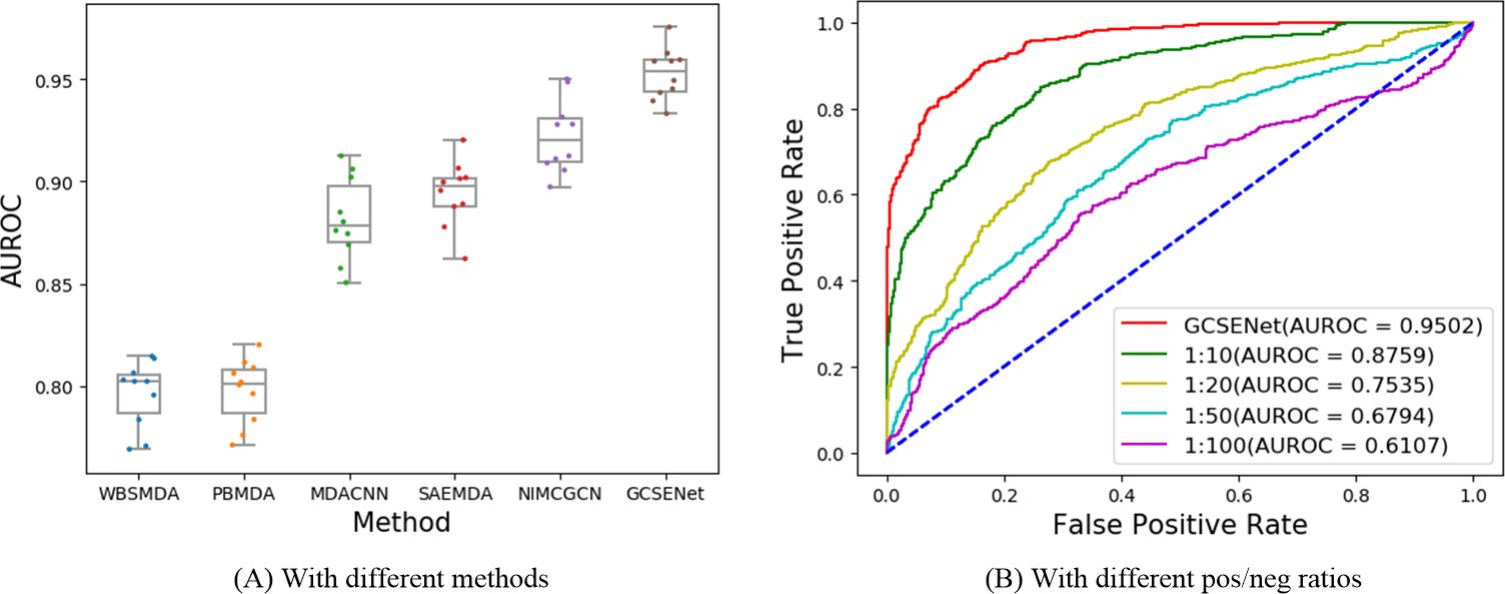
AUROC comparison of miRNA-disease in 10-fold cross-validation. (A) With different methods. (B) With different pos/neg ratios.

In order to confirm the advantages of our method on AUROC, a chart was presented in Fig 7A to show the AUROC value of the 10-fold cross-validation of various methods. It can be seen from this figure that the AUROC of our method on average was 0.9502+/-0.00016, which is superior to the best NIMCGCN reaching 0.9208+/-0.00032 among existing methods (WBSMDA, PBMDA, MDACNN, SAEMDA with average AUROC value of 0.8095+/-0.00027, 0.7990+/-0.00026, 0.8843+/-0.00042, 0.8997+/- 0.00026 respectively), and the minimum value of ours in miRNA-disease task was also higher compared to the mean value of the second-ranked method, which indicates that our method can lead to a more significant improvement to the value of AUROC than others.

### Performance comparison with different positive/negative ratios

In above training datasets, negative class examples are selected in equal proportion to positive examples. In reality, there may be hundreds negative miRNA-disease interactions for each positive one. So we set a different proportion of positive and negative examples (10:1, 20:1, 50:1, 100:1) to test the performance of our GCSENet model. The results in Fig 7B show that our association prediction is stable for the cases with different pos/neg ratios.

### Performance evaluation on predicting miRNA-phenotype associations

In addition to evaluating our GCSENet model on miRNA-disease association prediction, a further test was conducted to establish whether GCSENet can be applied to predict miRNA-phenotype associations. The study on the relationship between miRNA and disease phenotype can also reveal how miRNA affects human diseases.

Similar to the evaluation on miRNA-disease dataset, GCSENet was compared with five methods (WBSMDA [8], PBMDA [9], MDACNN [15], SAEMDA [10] and NIMCGCN [16]), with the results shown in Table 3. It was found out that GCSENet performed better than other methods in all metrics. For example, the average AUROC score of GCSENet was 0.9473, which is higher relative to the second best method NIMCGCN (the value is 0.9455). Similarly, GCSENet achieved the highest score in terms of AUPR, Precision, Recall and F1-score.

**Table 3:** Performance comparison with various methods for miRNA-phenotype association prediction.

methods	AUROC	AUPR	Precision	Recall	F1-score
WBSMDA	0.7023	0.7156	0.6834	0.7489	0.7147
PBMDA	0.7453	0.7197	0.6725	0.7614	0.7141
MDACNN	0.9429	0.9344	0.8667	0.8748	0.8704
SAEMDA	0.9212	0.9333	0.8864	0.8524	0.8691
NIMCGCN	0.9455	0.9367	0.8672	0.8501	0.8585
GCSENet	0.9473	0.9553	0.8721	0.8867	0.8793

**Fig 8:**
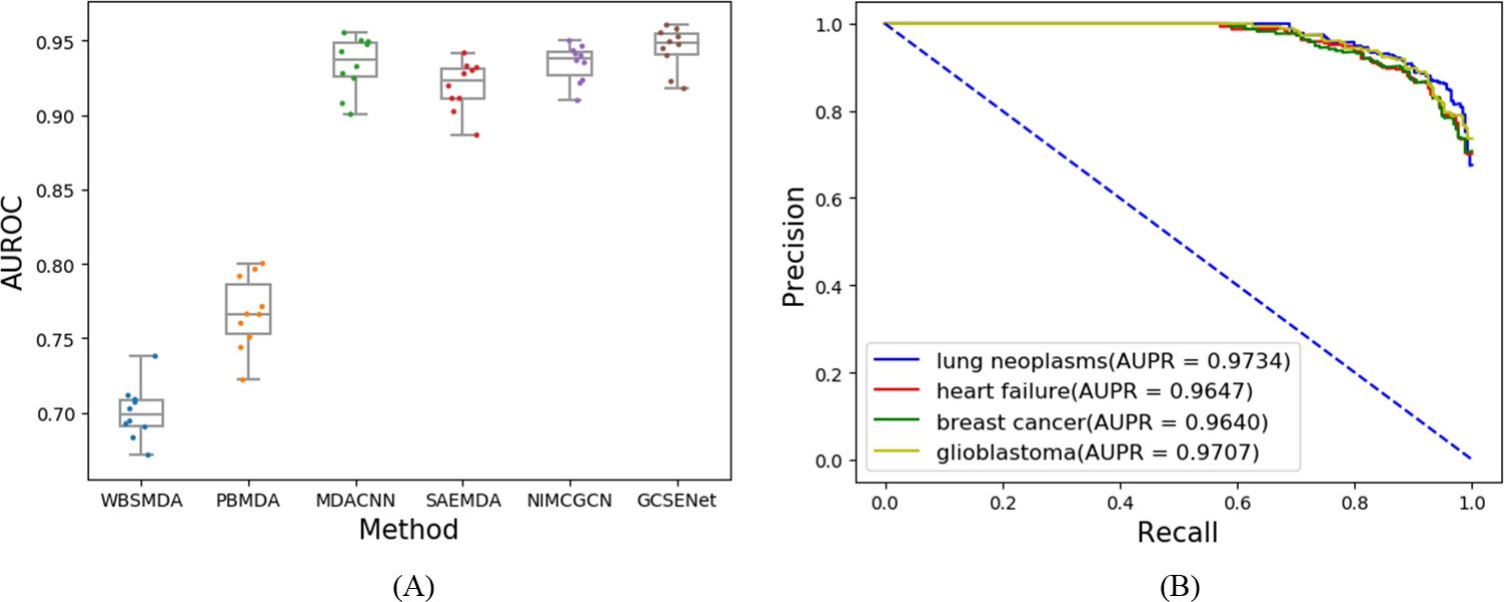
AUROC of miRNA-phenotype in 10-fold cross-validation (A), and Precision-Recall curve of lung neoplasms, heart failure, breast cancer and glioblastoma (B).

With regard to the prediction of miRNA-phenotype, a comparison was performed in AUROC between GCSENet and other methods, as shown in Fig 8A. Under the 10-fold cross-validation, the highest AUROC of GCSENet reached 0.96, the lowest was 0.92, and the average AUROC value was 0.9473+/-0.00028 (the average AUROC value of WBSMDA, PBMDA, MDACNN, SAEMDA, NIMCGCN are 0.7023+/-0.000267, 0.7453+/-0.001843, 0.9429+/-0.000348, 0.9212+/-0.000281 and 0.9455+/-0.00016, respectively). The lower quartile of GCSENet was higher compared to the median value of second-ranked NIMCGCN, while the upper quartile of GCSENet was also higher than the maximum value of NIMCGCN. From these metrics, it can be seen that GCSENet could produce a consistent performance in miRNA-phenotype prediction.

### Case study

**Fig 9:**
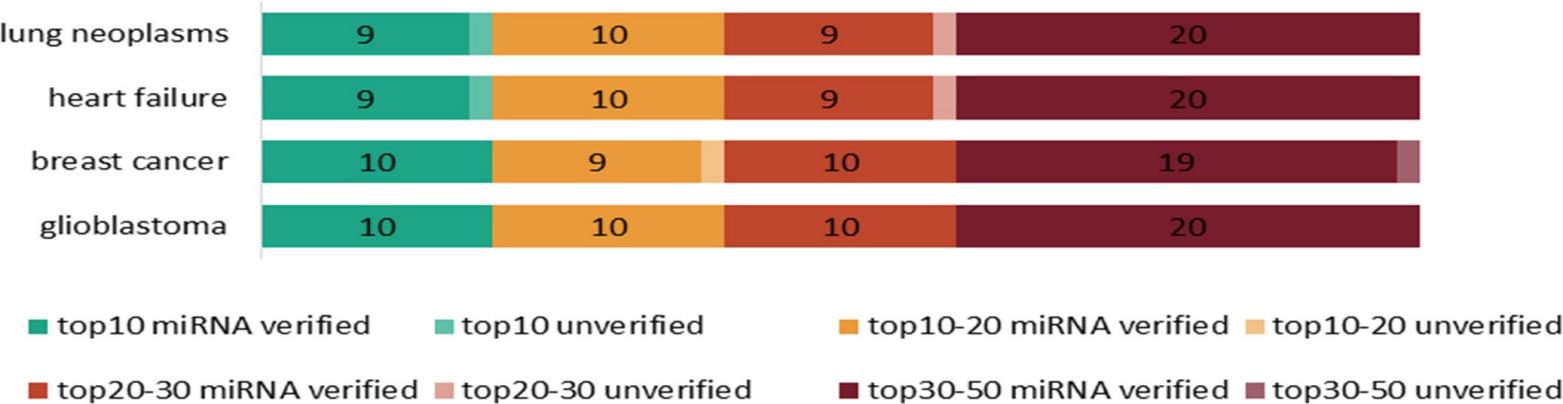
The number of predicted miRNAs verified in HMDD v3.0 by our model, including different top intervals.

Our prediction method was also applied for four specific examples (lung neoplasms, heart failure, breast cancer and glioblastoma). First of all, the relationship between these diseases and their related miRNAs in the heterogeneous network was removed during the training process. Then, GCSENet was used to predict the miRNAs associated with these diseases in the testing process. Finally, the result was compared with the existing relationship in HMDD v3.0. The AUPR curves of four diseases were shown in Fig 8B, the number of miRNA predicted by our method in different top intervals was listed in Fig 9.

There are 110 miRNAs directly related to lung neoplasms in HMDD v3.0. We know that 52 of 110 miRNAs, named ‘easy set’, have at least one known target gene in the protein network. 58 of 110 miRNAs, named ‘hard set’, have no known target gene in the protein network. It was found out that 106 miRNAs in the prediction results were obtained by our model, which are recorded in HMDD v3.0. It is no surprise that all miRNAs (52 of 52) in the ‘easy set’ are identified by GCSENet. In addition, 54 of 58 miRNAs in the ‘hard set’ are identified. When removing GCN, the feature weighting, the new feature component or SENet respectively in our model, 41 of 52 miRNAs (‘easy set’) are identified at most. In the ‘hard set’, only 15 of 58 miRNAs are identified. Consequently, there were only 56 miRNAs detected at most, which evidences the effectiveness of our complete GCSENet model.

For lung neoplasms, heart failure, breast cancer and glioblastoma, the precisions of our model are computed as 0.9209, 0.9230, 0.9243 and 0.9380, respectively. To determine whether GCSENet can effectively predict the miRNA-disease associations, the predicted miRNAs based on their prediction probabilities were ranked, which are shown in Tables 4–7. As for lung neoplasms, it can be found 9 out of the top 10, 19 out of the top 20, and 48 out of the top 50 predictions were manually confirmed in database HMDD v3.0. Also, 9 out of the top 10, 19 out of top 20, 28 out of top 30 and 48 out of the top 50 predicted miRNAs were verified as associated with heart failure in HMDD v3.0.

**Table 4:** Validation results of predicted associations for lung neoplasms as an unknown disease.

Rank	miRNAs	Evidence	Score	Rank	miRNAs	Evidence	Score
1	hsa-let-7a	HMDD v3.0	0.5249	26	hsa-let-7i	HMDD v3.0	0.5060
2	hsa-let-7b	HMDD v3.0	0.5245	27	hsa-mir-29c	HMDD v3.0	0.5053
3	hsa-mir-15a	HMDD v3.0	0.5242	28	hsa-mir-218	HMDD v3.0	0.5049
4	hsa-mir-29b	HMDD v3.0	0.5241	29	hsa-let-7f	HMDD v3.0	0.5031
5	hsa-mir-30e	HMDD v3.0	0.5239	30	hsa-mir-9	HMDD v3.0	0.5020
6	hsa-mir-155	HMDD v3.0	0.5238	31	hsa-mir-143	HMDD v3.0	0.4996
7	hsa-mir-29a	HMDD v3.0	0.5231	32	hsa-mir-106a	HMDD v3.0	0.4976
8	hsa-mir-16	unconfirmed	0.5224	33	hsa-mir-34c	HMDD v3.0	0.4972
9	hsa-mir-17	HMDD v3.0	0.5221	34	hsa-mir-17	HMDD v3.0	0.4966
10	hsa-mir-142	HMDD v3.0	0.5216	35	hsa-mir-1	HMDD v3.0	0.4963
11	hsa-mir-222	HMDD v3.0	0.5213	36	hsa-let-7g	HMDD v3.0	0.4959
12	hsa-mir-34a	HMDD v3.0	0.5209	37	hsa-mir-146b	HMDD v3.0	0.4953
13	hsa-mir-142	HMDD v3.0	0.5208	38	hsa-mir-214	HMDD v3.0	0.4947
14	hsa-mir-29c	HMDD v3.0	0.5207	39	hsa-mir-141	HMDD v3.0	0.4940
15	hsa-let-7e	HMDD v3.0	0.5205	40	hsa-mir-21	HMDD v3.0	0.4928
16	hsa-mir-34c	HMDD v3.0	0.5198	41	hsa-mir-181b	HMDD v3.0	0.4905
17	hsa-mir-146b	HMDD v3.0	0.5188	42	hsa-mir-101	HMDD v3.0	0.4883
18	hsa-let-7c	HMDD v3.0	0.5187	43	hsa-mir-125a	HMDD v3.0	0.4843
19	hsa-mir-146a	HMDD v3.0	0.5185	44	hsa-mir-34b	HMDD v3.0	0.4810
20	hsa-mir-30c	HMDD v3.0	0.5184	45	hsa-mir-133a	HMDD v3.0	0.4788
21	hsa-mir-100	HMDD v3.0	0.5175	46	hsa-mir-200b	HMDD v3.0	0.4763
22	hsa-let-7d	HMDD v3.0	0.5151	47	hsa-mir-200c	HMDD v3.0	0.4743
23	hsa-mir-15b	unconfirmed	0.5130	48	hsa-mir-182	HMDD v3.0	0.4727
24	hsa-mir-22	HMDD v3.0	0.5094	49	hsa-mir-191	HMDD v3.0	0.4712
25	hsa-mir-223	HMDD v3.0	0.5074	50	hsa-mir-126	HMDD v3.0	0.4703

**Table 5:** Validation results of predicted associations for heart failure as an unknown disease.

Rank	miRNAs	Evidence	Score	Rank	miRNAs	Evidence	Score
1	hsa-mir-29b	HMDD v3.0	0.5433	26	hsa-mir-132	HMDD v3.0	0.4960
2	hsa-mir-29a	HMDD v3.0	0.5402	27	hsa-mir-19a	HMDD v3.0	0.4956
3	hsa-mir-221	HMDD v3.0	0.5400	28	hsa-mir-15b	HMDD v3.0	0.4949
4	hsa-mir-21	HMDD v3.0	0.5362	29	hsa-mir-297	HMDD v3.0	0.4946
5	hsa-mir-195	HMDD v3.0	0.5349	30	hsa-let-7d	HMDD v3.0	0.4930
6	hsa-mir-19b	HMDD v3.0	0.5313	31	hsa-mir-212	HMDD v3.0	0.4911
7	hsa-mir-34a	HMDD v3.0	0.5299	32	hsa-mir-211	HMDD v3.0	0.4891
8	hsa-mir-29c	unconfirmed	0.5297	33	hsa-mir-210	HMDD v3.0	0.4880
9	hsa-mir-142	HMDD v3.0	0.5287	34	hsa-mir-30b	HMDD v3.0	0.4822
10	hsa-mir-155	HMDD v3.0	0.5276	35	hsa-mir-30a	HMDD v3.0	0.4803
11	hsa-let-7b	HMDD v3.0	0.5270	36	hsa-mir-372	HMDD v3.0	0.4781
12	hsa-mir-222	HMDD v3.0	0.5265	37	hsa-mir-520b	HMDD v3.0	0.4754
13	hsa-mir-92b	HMDD v3.0	0.5234	38	hsa-mir-526b	HMDD v3.0	0.4729
14	hsa-mir-18a	HMDD v3.0	0.5233	39	hsa-mir-381	HMDD v3.0	0.4718
15	hsa-let-7e	HMDD v3.0	0.5199	40	hsa-mir-382	HMDD v3.0	0.4710
16	hsa-mir-126	HMDD v3.0	0.5190	41	hsa-mir-429	HMDD v3.0	0.4694
17	hsa-mir-30e	HMDD v3.0	0.5188	42	hsa-mir-432	HMDD v3.0	0.4673
18	hsa-mir-192	HMDD v3.0	0.5138	43	hsa-mir-204	HMDD v3.0	0.4657
19	hsa-mir-17	HMDD v3.0	0.5088	44	hsa-mir-107	HMDD v3.0	0.4640
20	hsa-let-7c	HMDD v3.0	0.5038	45	hsa-mir-130a	HMDD v3.0	0.4595
21	hsa-mir-28	HMDD v3.0	0.5020	46	hsa-mir-139	HMDD v3.0	0.4560
22	hsa-mir-377	HMDD v3.0	0.5003	47	hsa-mir-199b	HMDD v3.0	0.4527
23	hsa-mir-650	HMDD v3.0	0.5000	48	hsa-mir-223	HMDD v3.0	0.4498
24	hsa-mir-101	unconfirmed	0.4998	49	hsa-mir-24	HMDD v3.0	0.4489
25	hsa-mir-22	HMDD v3.0	0.4996	50	hsa-mir-300	HMDD v3.0	0.4459

**Table 6:** Validation results of predicted associations for breast cancer as an unknown disease.

Rank	miRNAs	Evidence	Score	Rank	miRNAs	Evidence	Score
1	hsa-mir-429	HMDD v3.0	0.5372	26	hsa-let-7i	HMDD v3.0	0.4969
2	hsa-mir-141	HMDD v3.0	0.5367	27	hsa-mir-200a	HMDD v3.0	0.4964
3	hsa-mir-200a	HMDD v3.0	0.5341	28	hsa-let-7e	HMDD v3.0	0.4960
4	hsa-mir-200b	HMDD v3.0	0.5331	29	hsa-let-339	HMDD v3.0	0.4953
5	hsa-mir-29c	HMDD v3.0	0.5258	30	hsa-mir-9	HMDD v3.0	0.4949
6	hsa-mir-29b	HMDD v3.0	0.5255	31	hsa-mir-143	HMDD v3.0	0.4943
7	hsa-mir-196a	HMDD v3.0	0.5234	32	hsa-mir-106b	HMDD v3.0	0.4929
8	hsa-mir-200c	HMDD v3.0	0.5216	33	hsa-mir-34c	HMDD v3.0	0.4906
9	hsa-mir-7f	HMDD v3.0	0.5198	34	hsa-mir-15a	HMDD v3.0	0.4890
10	hsa-mir-335	HMDD v3.0	0.5194	35	hsa-mir-1	unconfirmed	0.4867
11	hsa-mir-125b	HMDD v3.0	0.5146	36	hsa-let-7g	HMDD v3.0	0.4821
12	hsa-let-98	unconfirmed	0.5144	37	hsa-mir-146b	HMDD v3.0	0.4791
13	hsa-mir-31	HMDD v3.0	0.5068	38	hsa-mir-214	HMDD v3.0	0.4777
14	hsa-mir-17	HMDD v3.0	0.5063	39	hsa-mir-488	HMDD v3.0	0.4750
15	hsa-mir-130	HMDD v3.0	0.5062	40	hsa-mir-429	HMDD v3.0	0.4727
16	hsa-let-373	HMDD v3.0	0.5057	41	hsa-mir-181b	HMDD v3.0	0.4717
17	hsa-mir-142	HMDD v3.0	0.5056	42	hsa-mir-10a	HMDD v3.0	0.4709
18	hsa-mir-192	HMDD v3.0	0.5049	43	hsa-mir-125a	HMDD v3.0	0.4688
19	hsa-let-101	HMDD v3.0	0.5046	44	hsa-mir-34b	HMDD v3.0	0.4663
20	hsa-mir-7c	HMDD v3.0	0.5045	45	hsa-mir-133a	HMDD v3.0	0.4656
21	hsa-mir-16	HMDD v3.0	0.5033	46	hsa-mir-181a	HMDD v3.0	0.4636
22	hsa-let-7d	HMDD v3.0	0.5022	47	hsa-mir-215	HMDD v3.0	0.4587
23	hsa-mir-146a	HMDD v3.0	0.4998	48	hsa-mir-24	HMDD v3.0	0.4557
24	hsa-mir-205	HMDD v3.0	0.4981	49	hsa-mir-222	HMDD v3.0	0.4525
25	hsa-mir-223	HMDD v3.0	0.4972	50	hsa-mir-203	HMDD v3.0	0.4491

**Table 7:** Validation results of predicted associations for glioblastoma as an unknown disease.

Rank	miRNAs	Evidence	Score	Rank	miRNAs	Evidence	Score
1	hsa-mir-21	HMDD v3.0	0.5433	26	hsa-mir-19a	HMDD v3.0	0.4988
2	hsa-mir-155	HMDD v3.0	0.5392	27	hsa-mir-143	HMDD v3.0	0.4973
3	hsa-mir-222	HMDD v3.0	0.5245	28	hsa-mir-145	HMDD v3.0	0.4971
4	hsa-mir-221	HMDD v3.0	0.5220	29	hsa-mir-16	HMDD v3.0	0.4966
5	hsa-mir-205	HMDD v3.0	0.5219	30	hsa-mir-10a	HMDD v3.0	0.4963
6	hsa-mir-451a	HMDD v3.0	0.5218	31	hsa-mir-504	HMDD v3.0	0.4958
7	hsa-mir-142	HMDD v3.0	0.5217	32	hsa-mir-99a	HMDD v3.0	0.4952
8	hsa-mir-206	HMDD v3.0	0.5215	33	hsa-mir-873	HMDD v3.0	0.4946
9	hsa-mir-34a	HMDD v3.0	0.5214	34	hsa-mir-885	HMDD v3.0	0.4938
10	hsa-mir-210	HMDD v3.0	0.5213	35	hsa-mir-425	HMDD v3.0	0.4918
11	hsa-mir-10b	HMDD v3.0	0.5212	36	hsa-mir-32	HMDD v3.0	0.4892
12	hsa-mir-23b	HMDD v3.0	0.5211	37	hsa-mir-22	HMDD v3.0	0.4882
13	hsa-mir-30b	HMDD v3.0	0.5210	38	hsa-mir-30a	HMDD v3.0	0.4836
14	hsa-mir-27b	HMDD v3.0	0.5207	39	hsa-mir-31	HMDD v3.0	0.4808
15	hsa-mir-193b	HMDD v3.0	0.5187	40	hsa-mir-128	HMDD v3.0	0.4785
16	hsa-mir-17	HMDD v3.0	0.5169	41	hsa-let-7d	HMDD v3.0	0.4759
17	hsa-mir-29a	HMDD v3.0	0.5159	42	hsa-mir-184	HMDD v3.0	0.4731
18	hsa-mir-27a	HMDD v3.0	0.5105	43	hsa-mir-218	HMDD v3.0	0.4727
19	hsa-mir-125b	HMDD v3.0	0.5088	44	hsa-mir-7	HMDD v3.0	0.4712
20	hsa-mir-25	HMDD v3.0	0.5087	45	hsa-mir-95	HMDD v3.0	0.4702
21	hsa-mir-29c	HMDD v3.0	0.5056	46	hsa-mir-200b	HMDD v3.0	0.4682
22	hsa-mir-302a	HMDD v3.0	0.5052	47	hsa-mir-149	HMDD v3.0	0.4660
23	hsa-mir-302b	HMDD v3.0	0.5045	48	hsa-let-7a	HMDD v3.0	0.4648
24	hsa-mir-302c	HMDD v3.0	0.5030	49	hsa-mir-224	HMDD v3.0	0.4606
25	hsa-mir-302d	HMDD v3.0	0.5003	50	hsa-mir-367	HMDD v3.0	0.4573

Similarly, GCSENet was applied to predict miRNAs associated with breast cancer. In the top 20 miRNAs with the highest prediction probability, it was found out that 19 miRNAs associated with breast cancer were included in the existing database. In the top 30 and 50, 29 and 48 miRNAs were verified as associated with breast cancer. For glioblastoma, it was found out that 20 miRNAs associated with glioblastoma in the top 20. In the top 30 and 50, 30 and 50 miRNAs were verified.

## Conclusion

The prediction of miRNA-disease association is of great significance to the study on the causes of diseases and drug treatment. In this paper, the GCN integrating with CNN and SENet was applied to identify the relationship between miRNA-disease and miRNA-phenotype. Firstly, GCN was used to extract the spatial structure feature of heterogeneous network, including disease, gene and miRNA. Then, a feature weight for each disease-gene association and miRNA-gene association was set to reflect this difference, and a new feature component was constructed by combining the different proportions of miRNA-gene association and disease-gene association. Subsequently, SENet was applied to determine the importance of each feature channel by means of the attention mechanism. Finally, the fully connected layer and softmax in CNN were set to predict the association between miRNA-disease and miRNA-phenotype. To demonstrate the advantages of our method, it was compared with other latest prediction methods. According to the experimental results, our method is superior to the existing methods. The source code is publicly available at https://github.com/Appleabc123/GCSENet.

At present, our model is also subject to some limitations, which need to be addressed in our further study. For example, the quality of the features extracted by graph convolution is vitally important, and it is dependent on the high-quality miRNA disease heterogeneous network. Thus, how to construct the high-quality heterogeneous network through biological and disease information is significant to identifying the miRNA-disease association. Besides, the miRNA-disease associations obtained from HMDD v3.0 database are far from sufficient, which means the corresponding associations can be explored from other datasets. Furthermore, the disease semantic similarity and miRNA functional similarity also have a problem with sparsity, which makes it more difficult to construct the heterogeneous network in an accurate way. By integrating the Gaussian interaction profile kernel similarity [33] inferred from the known miRNA-disease associations, it can be used to solve this problem to some extent. These are the issues that are supposed to be addressed in the further research work.

## Supporting information

S1 Data Data source. (DOCX)

S1 User Guide How to use our GCSENet. (DOCX)
