# Supplementary material for: GCSENet: A GCN, CNN and SENet ensemble model for microRNA-disease association prediction
Source: PLoS Comput Biol. 2021 Jun 3;17(6):e1009048. doi: 10.1371/journal.pcbi.1009048 (PMC8205154; doi:10.1371/journal.pcbi.1009048)
Supplement: S1 Data — (DOCX) [file pcbi.1009048.s001.docx]

**S1 Data**

Disease-miRNA associations:

HMDD3.0 <http://www.cuilab.cn/hmdd>

Disease-gene associations:

DisGeNet v7.0 <https://www.disgenet.org/downloads>

miRNA-gene associations:

miRWalk2.0 <http://mirwalk.umm.uni-heidelberg.de/>

disease-disease similarity network:

Mesh <http://www.ncbi.nlm.nih.gov/>

Gene-gene similarity network:

STRING <https://www.string-db.org/>

MiRNA similarity network:

MISIM http://www.cuilab.cn/files/images/cuilab/misim.zip

Test set:

Benchmark 2019 data <http://www.cuilab.cn/hmdd>

Phenotype-gene associations:

HPO database <https://hpo.jax.org/app/download/annotation>

miRNA-phenotype associations:

miRWalk2.0 <http://mirwalk.umm.uni-heidelberg.de/>
