# Supplementary material for: GCSENet: A GCN, CNN and SENet ensemble model for microRNA-disease association prediction
Source: PLoS Comput Biol. 2021 Jun 3;17(6):e1009048. doi: 10.1371/journal.pcbi.1009048 (PMC8205154; doi:10.1371/journal.pcbi.1009048)
Supplement: S1 User Guide — (DOCX) [file pcbi.1009048.s002.docx]

**S1 User Guide**

How to use our GCSENet: <https://github.com/Appleabc123/GCSENet>

1. GCSENet was implemented with python 3.6.4. To run GCSENet, you need these packages:

Matplotlib (3.1.1), (https://pypi.org/project/matplotlib/)

Networkx (2.5), (https://pypi.org/project/networkx/)

Tensorflow-gpu (1.4.0), (<https://pypi.tuna.tsinghua.edu.cn/simple/>tensorflow-gpu/)

Numpy (1.19.1), (https://pypi.org/project/numpy/)

Pandas (0.25.3), (https://pypi.org/project/pandas/)

Sklearn (0.20.3), (https://pypi.org/project/sklearn/)

Scipy (1.5.2), (<https://pypi.org/project/scipy/>)

2. The detailed information of GCSENet in

<https://github.com/Appleabc123/GCSENet>

There are two folders: ‘code’, ‘data’.

In the ‘code’ folder, it includes ‘1.Generate Feature by GCN’, ‘2.Feature Process’, ‘3.Train’, ‘4.Test’, which means the code to generate feature, the code to process feature, the code to train the model and the code to test the model, respectively.

In the ‘data’ folder, it contains ‘generate feature’, ‘process feature’, ‘CNN_SENet’, ‘Test’，which means the place to save the raw data, the place to save the feature components (miRNA-gene, disease-gene), the place to save the feature of miRNA-disease and the place to save the test dataset, respectively.

3. How to reproduce our results:

Download the code package from <https://github.com/Appleabc123/GCSENet>.

**Step1.** Get the feature vector (disease-gene, miRNA-gene)

**Set** the data_path in main.py, containing original data (d-d.csv, g-g.csv, d-g.csv,

disease_name.csv, gene_name.csv).


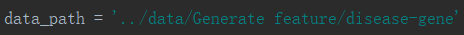


**Run** main.py to obtain the disease-gene vector.

The result includes the ‘disease-gene.csv’ file, which is saved in ‘../data/process_ feature’ folder.


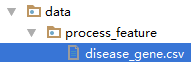


Similarly,

**Set** the data_path in main.py, containing original data (m-m.csv, g-g.csv, m-g.csv, miRNA_name.csv, gene_name.csv).


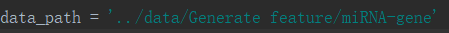


**Run** main.py to obtain the miRNA-gene vector.

The result includes the ‘miRNA-gene.csv’ file, which is saved in ‘../data/process_ feature’ folder.


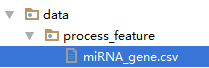


**Step2.** Get the weighted feature (disease-miRNA) and label

**Run** process_feature.py to obtain the disease-miRNA vector.

The result includes two files with ‘disease-miRNA.csv’ and ‘label.csv’, which are saved in the ‘../data/CNN_SENet’ folder.


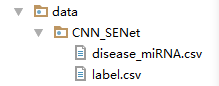


**Step3.** Train the network

**Run** CS_train.py to train the model.


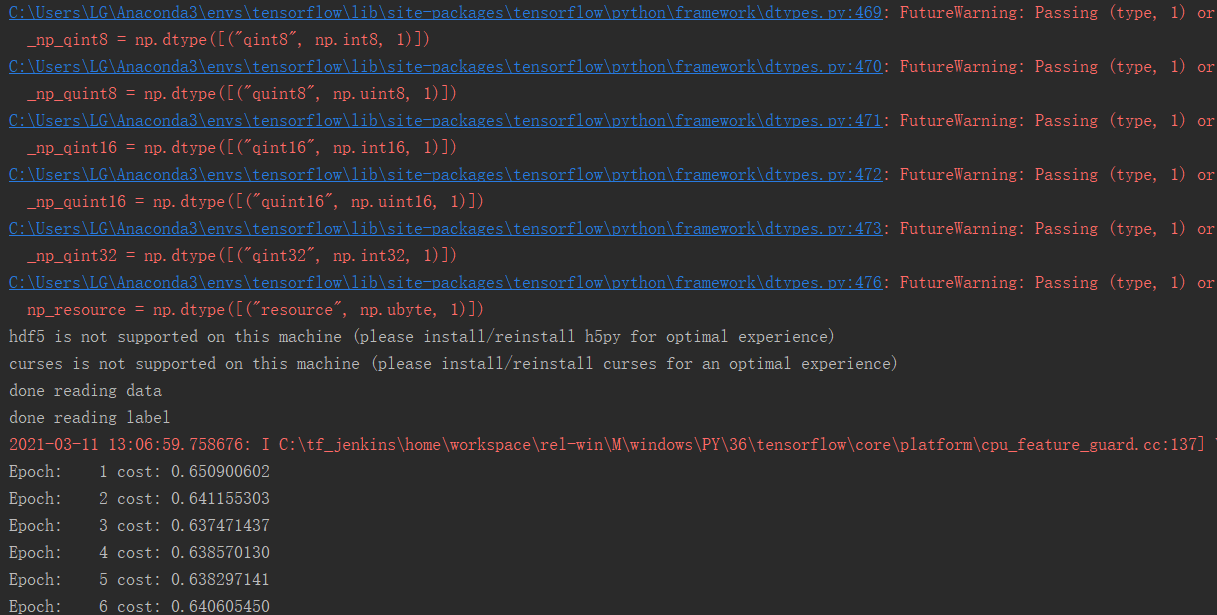


**Step4.** Test the benchmark2019 set to get the AUROC, AUPR, Precision, Recall, F1-score

**Run** CS_test.py to test the model.


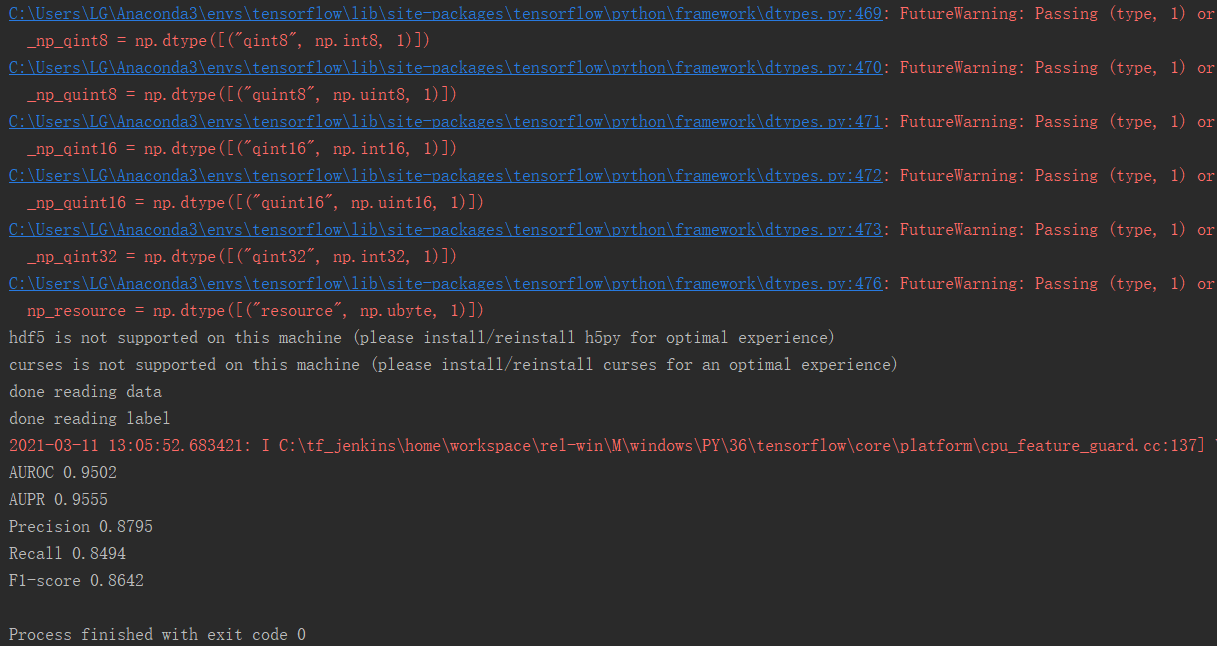


4. How to use the framework on your interested datasets (disease, gene, miRNA) as training or test datasets?

**Step 1.** Generate the feature vector of miRNA-gene, disease-gene

**(1). Put** the interested data (d-d.csv, g-d.csv, g-g.csv, disease_name.csv, gene_name.csv) in the ‘../data/Generate feature/disease-gene’ folder, the interested data (g-g.csv, g-m.csv, m-m.csv, gene_name.csv, miRNA_name.csv) in the ‘../data/Generate feature/miRNA-gene’ folder, respectively.


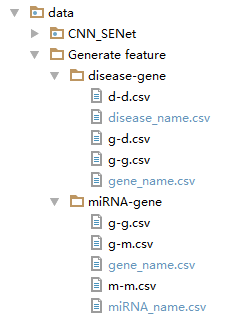


**(2). Set** some parameters in main.py:

data_path = ‘../data/Generate feature/disease-gene’.

#setting the data directory as the directory where you save the raw data (disease-gene, miRNA-gene).


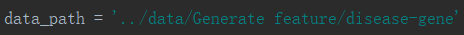


save_path = ‘../data/process_feature’.

#the directory is the place to save the feature vectors of disease-gene and miRNA-gene, which will be used in the following step 2.


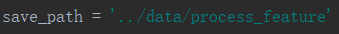


**(3).** **Run** ‘main.py’ to get the feature files of disease-gene and miRNA-gene, which will be saved in the given path.


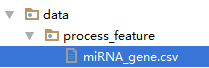


**Step 2.** Process the feature vector to get the feature of miRNA-disease and label

**(1). Put** the positive sample (pos.txt) and negative sample (neg.txt) in the ‘../data/process_feature’.


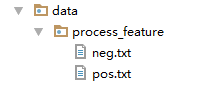


**(2). Set** some parameters in process_feature.py:

input_postive = ‘../data/process_feature/pos.txt’.

#the directory is used for saving the positive sample file from the test dataset. In our GCSENet, the positive samples are from benchmark2019.

input_negative = ‘../data/process_feature/neg.txt’.

#the directory is used for saving the negative sample file. The negative samples in the test dataset are not in the dataset generating positive samples.

Output = ‘../data/CNN_SENet’.

#the directory is the path where you save the output files of miRNA-disease feature and label.


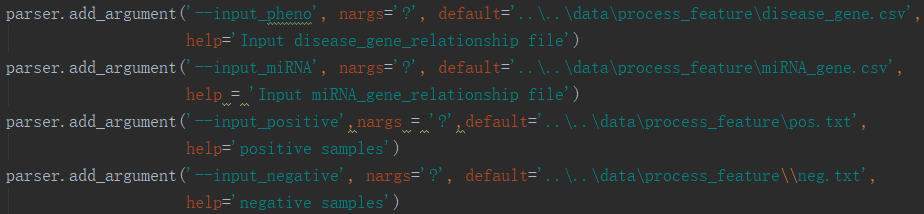


**(3). Run** ‘process_feature.py’ to get the miRNA-disease feature (disease -miRNA.csv) and label (label.csv), which will be used in the following step 3.


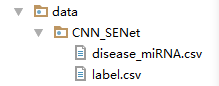


**Step 3.** Train the model

**Run** CS_train.py to train the model.

**Step 4.** Test the model

**Run** CS_test.py to get the AUROC, AUPR, Precision, Recall, F1-score.
